# An Exploratory Research to Evaluate the 30 Most Common Pulmonary Embolism Drugs in the Food and Drug Administration Adverse Event Reporting System

**DOI:** 10.1111/crj.70054

**Published:** 2025-06-05

**Authors:** Hang Chen, Yiming Shen, Yinyu Mu, Shuguang Xu, Shimo Shen, Weiyu Shen, Zeyang Hu, Hongxiang Li, Keyue Qiu, Jiaheng Zhang, Zhe Chen, Guodong Xu

**Affiliations:** ^1^ Department of Thoracic Surgery, The Affiliated Lihuili Hospital Ningbo University Ningbo Zhejiang Province China; ^2^ Department of Otology and Skull Base Surgery, Eye Ear Nose and Throat Hospital Fudan University Shanghai China; ^3^ Department of Laboratory, The Affiliated Lihuili Hospital Ningbo University Ningbo Zhejiang Province China; ^4^ Department of Respiratory, The Affiliated Lihuili Hospital Ningbo University Ningbo Zhejiang Province China; ^5^ Medical School Ningbo University Ningbo Zhejiang Province China

**Keywords:** adverse events, drugs, FDA adverse event reporting system, molecular targeted agents, pulmonary embolism

## Abstract

**Backgrounds:**

Pulmonary embolism (PE) is a common disease and a common cause of death. However, it is currently unclear which clinically common drugs can lead to PE.

**Methods:**

We collected, organized, and analyzed reports from the first quarter of 2018 to the fourth quarter of 2022. We performed disproportionality analysis algorithms to calculate reporting odds ratio (ROR), which could quantify the signal values of different adverse events (AEs).

**Results:**

We have screened a total of 3091 drugs, with AE containing “PE” and calculated the ROR signal values of the top 30 drugs reported and ranked them. TESTIM (ROR = 32.03[28.77–35.66]), BARICITINIB (ROR = 23.48[20.55–26.83]), and NUVARIANG (ROR = 19.89[17.13–23.10]) are the drugs with the strongest correlation with PE. In addition, among the 30 drugs with the strongest correlation with PE, most of which are Biologics & Immunomodulators. Therefore, when using these 30 drugs, it is necessary to be alert to the possible risk of PE.

**Conclusion:**

In our study, we filtered 30 common drugs that could cause PE through the FAERS public database, which provides theoretical support for drug selection in the treatment of malignant tumors and IMID.

## Graphical abstract

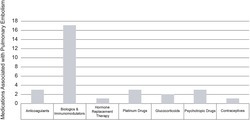

## 1 Introduction

Although the incidence rate of cardiovascular diseases in the United States decreased by about 4% year by year during the decade from 2000 to 2011, the downward trend has gradually slowed down in recent years [1, 2, 3]. Pulmonary embolism (PE) means that the embolus may lead to acute life‐threatening right heart failure by blocking the pulmonary artery [4] and is the third most common cause of cardiovascular death worldwide after stroke and heart attack [5]. The common causes of PE are deep vein thrombosis (DVT) falling off to the pulmonary artery, in situ thrombosis, fat embolism, and amniotic fluid embolism, of which DVT of lower limbs is the most common cause [6]. If not treated in a timely manner or improperly, it may also cause serious long‐term complications, namely, chronic thromboembolic pulmonary hypertension (CTEPH) [7]. In recent years, with the breakthrough of local thrombolysis, mechanical extraction devices, hemodynamic support devices (such as extracellular membrane oxygenation), and surgical embolectomy, the treatment of PE has also developed [8]. However, the hospital all‐cause mortality rate of high‐risk patients in the United States is as high as 52.2% [9]. Therefore, reasonable stratification and early prevention are of great significance to reduce the incidence of PE and improve the life quality of patients [10].

As one of the diseases with the highest mortality rate of cancer‐related vascular disease, PE is often caused by multiple factors such as congestion, endothelial damage, and hypercoagulability in tumor patients [11, 12]. Patients with lung cancer, ovarian cancer, breast cancer, pancreatic cancer, and liver cancer are more likely to have PE than patients with other types of solid tumors. Furthermore, patients with distant metastasis have a higher risk of PE [13]. In recent years, breakthroughs in targeted therapy and immunotherapy have greatly improved the overall survival (OS) and quality of life of patients with malignant tumors [14, 15]. However, there are also relevant studies that show that targeted drugs and immune drugs have the risk of causing PE [16, 17]. Therefore, patients with malignant tumors need to be more alert to the occurrence of PE when receiving Biologics & Immunomodulators including molecular targeted drugs and immune drugs.

Immune mediated inflammatory disease (IMID) is a group of complex inflammatory diseases caused by immune pathway disorders, which can involve any system in the human body, which are generally closely related to genetics and environment [18, 19]. In recent years, relevant studies have also found that patients with IMIDs, including inflammatory bowel disease, Crohn's disease, ulcerative colitis, and rheumatoid arthritis, psoriasis, have a significantly increased risk of VTE [20]. Similar to malignant tumors, IMIDs are often treated with biologic therapy [15, 21]. Moreover, many studies have proposed that the use of molecular targeted agents will also lead to a significant increase in the risk of PE [22, 23]. However, there is no research focus on comparing the risk level of PE with different molecular targeted agents.

In the present study, we hope to quantify the correlation between different drugs and PE through the FAERS public database and screen out the top 30 drugs that are most likely to cause PE in clinic, of which 18 are molecular targeted agents.

## 2 Materials and Methods

### 2.1 Data Download

This study is a retrospective study aimed at screening the top 30 drugs that lead to “PE”. We downloaded the data files from the FAERS database (https://fis.fda.gov/extensions/FPD‐QDE‐FAERS/FPD‐QDE‐FAERS.html), including patient demographic and administrative information (DEMO), drug information (DRUG), coded for the adverse events (REAC), patient outcomes (OUTC), report sources (RPSR), therapy start dates and end dates for reported drugs (THER), indications for drug administration (INDI), and deleted cases. In order to more accurately filter to the drugs that lead to PE (PT 10037377), the data profiles in the past 5 years (from the first quarter of 2018 to the fourth quarter of 2022) were utilized for the subsequent analysis. All data downloaded from the FDA website are imported into MySQL 8.0 for further analysis, and then duplicate data are deleted and processed. Subsequently, the AEs in the FAERS database were annotated by the Medical Dictionary for Regulatory Activities 26.0 (MedDRA) [24].

### 2.2 Signal Analysis

The reporting odds ratio (ROR) value represents the correlation between the drug and the target AE, and the larger the ROR value, the stronger the correlation between the two [25]. We set as “PE” and subsequently calculate the ROR values and corresponding 95% confidence interval (CI) of drugs which rank the top 30 causing PE by using the following formula:

$$\mathrm{ROR}=\frac{\left(a/c\right)}{\left(b/d\right)}$$

Then, we reordered the 30 drugs based on the ROR values and corresponding 95% CI and classify the types of drugs into several categories, including Anticoagulants, Biologics & Immunomodulators, Hormone Replacement Therapy, Platinum Drugs, Glucocorticoids, Psychotropic Drugs, and Contraceptives, and count the number, respectively.

## 3 Results

**FIGURE 1:**
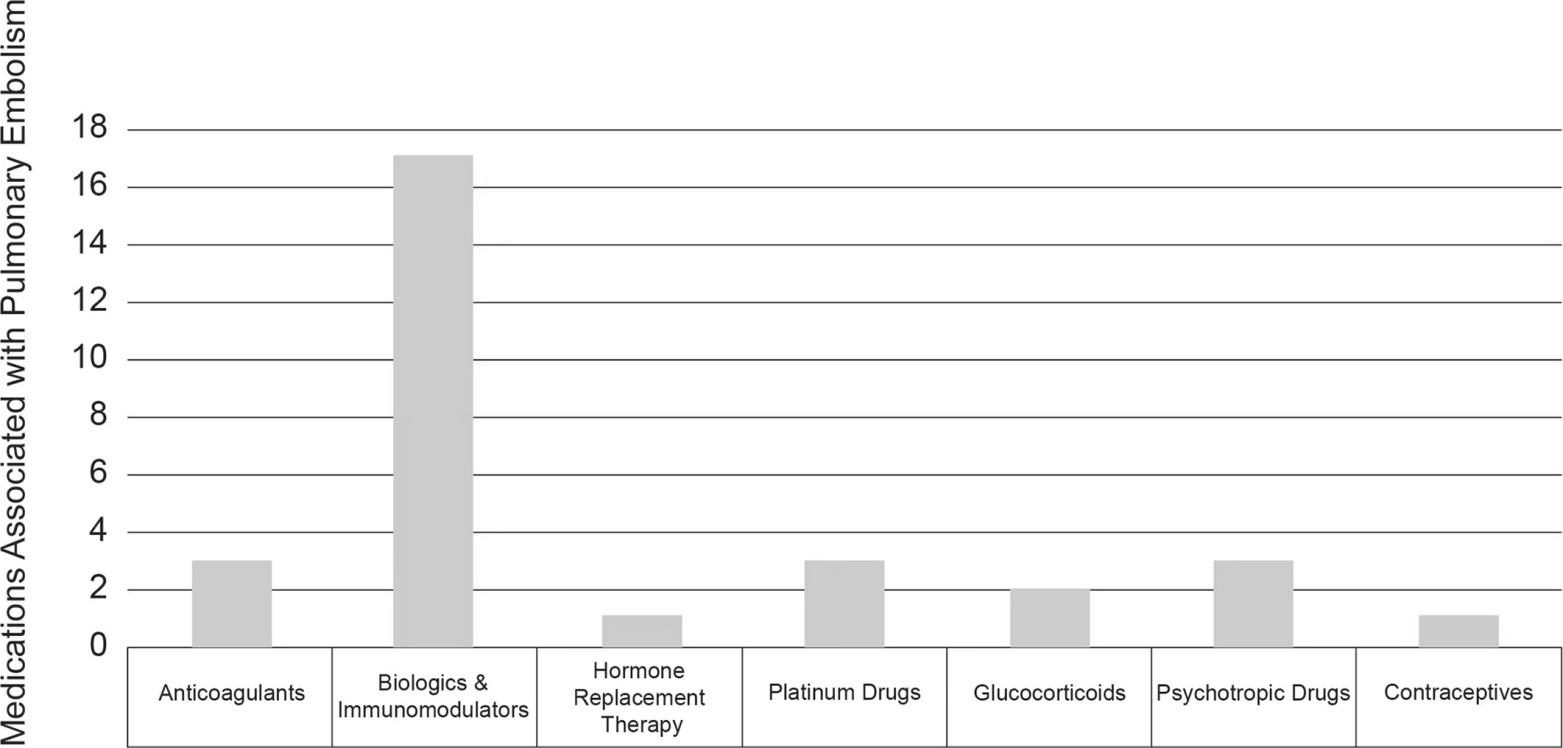
The drug categories of the 30 drugs that most commonly cause pulmonary embolism in the FAERS database.

We obtained a total of 3091 previously reported drugs causing PE and calculated the ROR values and corresponding 95% CI of the top 30 drugs reported (Table 1). Subsequently, we reordered the calculated ROR values and obtained the Top 30 drugs associated with PE (Table 2). We have classified the classifications of 30 drugs obtained, including Biologics & Immunomodulators (BARICITINIB, LETROZOLE, CAPECITABINE, TAGRISSO, LENVIMA, REVLIMID, TRASTUZUMAB, OFEV, POMALYST, BEVACIZUMAB, CABOMETYX, NIVOLUMAB, KEYTRUDA, RITUXIMAB, RINVOQ, XELJANZ, IBRANCE), Anticoagulants (APIXABAN, XARELTO, PRADAXA), Hormone Replacement Therapy (TESTIM), Platinum Drugs (CISPLATIN, CARBOPLATIN, OXALIPLATIN), Glucocorticoids (PREDNISONE, PREDNISOLONE), Psychotropic Drugs (OLANZAPINE, QUETIAPINE, DUODOPA), and Contraceptives (NUVARING), and calculated their percentages (Figure 1). Therefore, we found that the vast majority of the top 30 drugs associated with PE are Biologics & Immunomodulators, accounting for approximately 56.67%. This means that it is necessary to be vigilant about the occurrence of PE when receiving the aforementioned Biologics & Immunomodulators.

**TABLE 1:** Top 30 medications associated with pulmonary embolism from the FAERS arranged by frequency, January 2018 to December 2022.

Ranking	Medication	Frequency
1	REVLIMID	1053
2	XARELTO	776
3	APIXABAN	774
4	XELJANZ	340
5	RITUXIMAB	240
6	NIVOLUMAB	235
7	PRADAXA	225
8	QUETIAPINE	216
9	IBRANCE	212
10	OLANZAPINE	211
11	POMALYST	204
12	CARBOPLATIN	181
13	TESTIM	179
14	NUVARING	177
15	KEYTRUDA	146
16	CABOMETYX	146
17	LENVIMA	136
18	LETROZOLE	136
19	OXALIPLATIN	131
20	CAPECITABINE	127
21	BARICITINIB	126
22	OFEV	116
23	DUODOPA	113
24	RINVOQ	111
25	PREDNISONE	111
26	CISPLATIN	110
27	PREDNISOLONE	108
28	BEVACIZUMAB	98
29	TAGRISSO	89
30	TRASTUZUMAB	87

**TABLE 2:** Top 30 medications associated with pulmonary embolism from the FAERS ranked by ROR, January 2018 to December 2022.

Ranking	Medication	ROR	ROR lower bound, 95% CI	ROR upper bound, 95% CI
1	TESTIM	32.03	28.77	35.66
2	BARICITINIB	23.48	20.55	26.83
3	NUVARING	19.89	17.13	23.10
4	APIXABAN	17.58	16.00	19.30
5	XARELTO	8.05	7.49	8.65
6	PRADAXA	6.48	5.69	7.39
7	CISPLATIN	5.79	4.84	6.93
8	OLANZAPINE	4.16	2.16	8.01
9	LETROZOLE	4.12	3.64	4.67
10	CAPECITABINE	3.72	3.08	4.49
11	TAGRISSO	3.72	3.08	4.49
12	QUETIAPINE	3.25	2.89	3.66
13	CARBOPLATIN	3.20	2.78	3.67
14	LENVIMA	3.08	2.69	3.53
15	OXALIPLATIN	3.05	2.61	3.57
16	REVLIMID	3.00	2.82	3.19
17	TRASTUZUMAB	2.62	2.20	3.12
18	OFEV	2.61	2.25	3.04
19	POMALYST	2.48	2.18	2.82
20	BEVACIZUMAB	2.44	2.09	2.86
21	CABOMETYX	2.44	2.12	2.81
22	NIVOLUMAB	2.36	2.11	2.64
23	KEYTRUDA	2.12	1.83	2.45
24	PREDNISONE	1.72	1.44	2.07
25	PREDNISOLONE	1.61	1.38	1.86
26	RITUXIMAB	1.54	1.40	1.69
27	RINVOQ	1.52	1.27	1.83
28	XELJANZ	1.51	1.38	1.65
29	IBRANCE	1.39	1.23	1.57
30	DUODOPA	1.37	1.15	1.64

## 4 Discussion

In this study, 30 drugs with the strongest correlation with PE were found through FAERS database, more than half of which were Biologics & Immunomodulators, providing theoretical guidance for drug use and prevention of PE. When patients receive the above drug treatment, they need to be vigilant about the risk of PE, in which anticoagulant drugs can be used to prevent if the risk of thrombosis in patients is high. Next, I will introduce the top 30 drugs related to PE in detail. TESTIM is the drug with the strongest correlation with PE among the 30 drugs. Hazem et al. proposed that TESTIM can cause erythrocytosis and platelet agglutination, thus causing PE [26]. As the first available contraceptive vaginal ring approved by the FDA, NUVARIANG contains estrogen and progesterone, which has been reported to cause PE just like oral contraceptives [27]. In conclusion, we found that sex hormones, including TESTIM and the NUVARIANG, can increase the incidence of PE both.

Biologics & Immunomodulators is the most numerous classification among the top 30 drugs related to PE. Biologics & Immunomodulators are widely used in targeted therapy, immunotherapy, and IMID of malignant tumors, among which malignant tumors and IMID are already high risk factors of PE. Therefore, PE is likely to be induced when the above 30 drugs are used to treat malignant tumors and IMID, and anticoagulant can be used in advance for prevention when necessary. For instance, as two of four Janus kinase inhibitors (JAKis) approved for marketing in the United States for the treatment of rheumatoid arthritis (RA), the complications of PE caused by BARICITINIB and XELJANZ have attracted more and more attention [28]. Besides, the third‐generation aromatase inhibitors, including LETROZOLE, are twice as likely to have PE as patients using other drugs [29]. Additionally, patients who have not received chemotherapy for advanced breast cancer are more likely to have PE when receiving CAPECITALINE [30]. Furthermore, in the phase II multicenter clinical trial (NCT02094261), it was found that the most common adverse reaction of TAGRISSO in the treatment of non–small cell lung cancer was PE [31]. Moreover, a patient with anaplastic thyroid cancer was reported to have PE when receiving LENVIMA [32]. As one of the immunomodulators for the treatment of multiple myeloma (MM), the incidence of PE in patients receiving REVLIMID is significantly higher [33]. In addition, Lin et al. reported that two breast cancer patients with overexpression of HER‐2 had PE when receiving TRASTUZUMAB [34]. Additionally, PE is a common complication in the treatment of unresectable epithelioid malignant pleural Mesothelioma by OFEV [35]. Similar to REVLIMID, when POMALYST is used as a immunosuppressive drug to treat patients with MM, the risk of PE in patients is further increased [36]. BEVACIZUMAB is a monoclonal antibody against vascular endothelial growth factor (VEGF), which is related to hemoptysis and PE, especially in patients with pulmonary squamous cell carcinoma [37]. According to the clinical study reported by David et al., we found that patients with recurrent Glioblastoma had PE when receiving CABOMETYX [38]. Moreover, Liao et al. reported that a patient with adenocarcinoma of the lung developed acute PE and severe pneumonia after receiving NIVOLUMAB immunotherapy [39]. Furthermore, Tamara et al. recruited 228 patients with Melanoma and found that the incidence of PE was significantly increased after KEYTRUDA immunotherapy [40]. Further, Sonia et al. reported a 36‐year‐old female patient who died after PE when using RITUXIMAB to treat antiphospholipid syndrome, systemic lupus erythematosus and scleroderma [41]. Additionally, patients with active Ulcerative colitis experienced PE when receiving RINVOQ [42]. According to Lorenzo et al., when patients with hormone receptor positive metastatic breast cancer receive IBRANCE treatment, more patients tend to have PE [43]. In conclusion, as for patients with malignant tumors and immune inflammation, the probability of PE is significantly increased when the above targeted drugs are used for treatment. It is extremely important to include the aforementioned drugs in the thrombus risk assessment list.

Generally speaking, Biologics & Immunomodulators are mainly used for patients with various malignant tumors. The pathological and physiological mechanisms underlying the significantly increased incidence of PE in patients receiving Biologics & Immunomodulators may involve the following aspects:Firstly, patients with cancer are often elderly and weak, with less physical activity. Some patients even stay in bed for a long time, leading to stagnant blood flow and significantly increasing the incidence of thrombosis. Secondly, cancer patients often need to take medication continuously for several days during treatment. In order to avoid repeated needle insertion, most require the placement of central venous catheters, such as PICC (peripherally inserted central catheter) and intravenous infusion port. It is a foreign substance for blood vessels, which can easily cause clotting substances in the blood to accumulate on their surface, leading to thrombosis and detachment, which can easily cause PE. Thirdly, the treatment methods used during treatment, such as chemotherapy, radiotherapy, and surgery, can accelerate the formation of blood clots to varying degrees.

Among the 30 drugs, there are also three drugs for PE, namely, APIXABAN, XARELTO, and PRADAXA. Due to the fact that patients who use these three anticoagulant drugs are already high‐risk patients for PE, we usually consider PE as a complication of thrombolysis failure of these three drugs. For instance, there are also relevant studies reported that patients with PE after using APIXABAN. Furthermore, when combined with potent inducers of CYP3A4 and P‐glycoprotein such as Rifampicin, Phenytoin, Phenobarbital, and St. John's wort, the blood concentration of APIXABAN may also decrease. Therefore, the efficacy of APIXABAN is significantly reduced in patients receiving the above‐mentioned drug treatment [44].

As the first anticoagulant approved by FDA for acute and continuous treatment of PE, XARELTO has also been reported to cause PE. It is important to note that the bioavailability of XARELTO may be affected if not taken as directed, which typically involves taking the 15‐ or 20‐mg tablets before a meal [45]. Studies have suggested that compared with Warfarin, the risk of major bleeding with PRADAXA, a reversible direct thrombin inhibitor, may be lower, although the risk of VTE and PE may vary. PRADAXA is packaged in water‐soluble film, which protects the capsules from moisture and light; therefore, unauthorized changes in dosage or premature discontinuation of therapy should be avoided to prevent potential arterial embolism events. Anecdotal reports suggest that improper storage, such as transferring capsules to an unprotected container, may compromise the medication's efficacy and pose a risk of adverse events, including stroke. However, these reports require further investigation and substantiation with clinical data [46].

The target audience of platinum drugs is malignant tumor patients, who have a higher risk of basic thromboembolism.

Therefore, CISPLATIN, CARBOPLATIN, and OXALIPLATIN are more likely to cause PE in platinum drugs. As the earliest discovered chemotherapy drug, the significant side effects of CISPLATIN pose a huge challenge for effective treatment of cancer patients [47]. However, the PE caused by CISPLATIN has gradually attracted people's attention in recent years [48]. Patients receiving CARBOPLATIN chemotherapy may increase the incidence of PE [49]. When patients with advanced pancreatic cancer receive OXALIPLATIN, PE is a relatively related complication [50].

Glucocorticoid regulates many physiological processes and is the main drug for treating inflammatory diseases, autoimmune diseases and cancer, but it is also accompanied by many side effects, among which the more common is to increase the risk of incidence rate and mortality of cardiovascular diseases [51].

Recently, some studies have also proved that exogenous glucocorticoids can promote the synthesis and secretion of vascular hemophilia factor and plasmin activator inhibitor‐1, suggesting that they directly activate blood coagulation and inhibit glycolytic enzyme [52, 53]. Fred et al. reported a phase III clinical trial, which found that the incidence of PE in prostate cancer patients was significantly increased after receiving PREDNISONE treatment [54]. It has also been reported that PREDNISOLONE may cause PE in the treatment of filariasis [55].

There is no clear explanation for the increased risk of venous thrombosis among antipsychotic drug users. However, scholars have proposed several hypotheses, including increased platelet aggregation [56], anticardiolipin antibodies [56], increased venous stasis during sedation [56], increased adrenaline secretion during acute psychosis [57], and hyperhomocysteinemia [58].

In this study, we found that three antipsychotic drugs were closely related to the occurrence of PE, including OLANZAPINE, QUETIAPINE, and DUODOPA. For example, relevant research shows that OLANZAPINE may cause PE when it is used to treat psychotic patients for a long time, even at a low dose [59]. According to the case report recorded by Thomas, we found that patients with psychosis developed PE after receiving QUETIAPINE treatment [60]. As a therapeutic drug for patients with Parkinson's syndrome, DUODOPA is also a common cause of PE, which is also a common cause of death for patients with Parkinson's syndrome [61].

We are the first study to quantify the risk value of PE and rank the signal values of the top 30 drugs, which provides a theoretical basis for avoiding PE in clinical medication. However, there are some limitations in my research. Above all, our research is limited to the analysis of public databases and has not been proven to be accurate through clinical trials. Secondly, we only calculated the AE signal value of the first 30% of the reported number of drugs, but not all drugs, which inevitably missed some drugs that are not only commonly used but also easy to cause PE. Thirdly, the factors that cause PE include various clinicopathological characteristics, including age, gender, and basic diseases. Our analysis does not specifically distinguish these factors, which will be improved later.

In our study, we filtered 30 common drugs that could cause PE through the FAERS public database, which provides theoretical support for drug selection in the treatment of malignant tumors and IMID.

## Consent

The authors have nothing to report.

## Author Contributions

Hang Chen and Guodong Xu contributed to the conception of the study. Yiming Shen, Yinyu Mu, Shuguang Xu, Shimo Shen, and Weiyu Shen performed the SAS software and contributed significantly to the analysis and manuscript preparation. Zeyang Hu, Hongxiang Li, Keyue Qiu, Jiaheng Zhang, and Weiyu Shen performed the data analyses and wrote the manuscript. Zhe Chen helped to perform the analysis and contributed constructive discussions.

## Ethics Statement

The authors have nothing to report.

## Conflicts of Interest

The authors declare no conflicts of interest.

## Data Availability

Data sharing is not applicable to this article as no new data were created or analyzed in this study.
